# Exudative glomerulonephritis associated with acute leptospirosis in dogs

**DOI:** 10.1177/03009858231207020

**Published:** 2023-10-29

**Authors:** Monika Hilbe, Horst Posthaus, Giulia Paternoster, Simone Schuller, Michelle Imlau, Hanne Jahns

**Affiliations:** 1 Vetsuisse Faculty, Zurich, Switzerland; 2 University of Bern, Bern, Switzerland; 3 University of Zurich, Zurich, Switzerland; 4 University College Dublin, Dublin, Ireland

**Keywords:** dog, immunohistochemistry, interstitial nephritis, kidney, *Leptospira*, leptospirosis, PCR, Warthin and Starry stain

## Abstract

In the past 20 years in Switzerland, dogs with suspect acute leptospirosis frequently showed severe glomerular changes that had not been previously reported. These features were characterized by abundant extravasated erythrocytes and fewer neutrophils accompanied by marked fibrin exudation into the urinary space that was interpreted as an exudative glomerulonephritis (GN). This retrospective study describes this significant glomerular pathological change and investigates the association with leptospirosis. Tissues from 50 dogs with exudative GN, retrieved from 2 pathology archives in Switzerland were reviewed using hematoxylin and eosin, periodic acid-Schiff, phosphotungstic acid-hematoxylin, and Warthin and Starry stains. Clinical and postmortem data were collected for each case. Immunohistochemistry (IHC) and/or polymerase chain reactions were used as confirmatory tests for leptospirosis. While all 50 cases had clinical and pathological features supporting a diagnosis of leptospirosis, 37 cases were confirmed for the disease. Using a LipL32 antibody in addition to the OMV2177 antibody raised against the lipopolysaccharide of *Leptospira interrogans* serovar Copenhageni increased the detection rate of *Leptospira* by IHC in exudative GN from 24% to 62%. Signalment, seasonality, clinical signs, blood results, and pathological changes in dogs with exudative GN were similar to those reported for dogs without GN and confirmed infection by *Leptospira* spp.. Exudative GN was common among Swiss dogs with leptospirosis where it caused acute severe disease. Leptospirosis should be considered as a cause of this new pathologic feature by the pathologist. The pathogenesis remains unclear, but involvement of a geographic-specific serovar with unique virulence factors is suspected and warrants further investigation.

Leptospirosis is a zoonosis with worldwide distribution that affects a large variety of mammalian species.^17^ It is caused by pathogenic spirochetes of the genus *Leptospira* that consist of 35 species classified into 3 phylogenetic clusters.^40^ Geographic differences in the distribution of serovars are marked and the three main pathogenic species with global distribution are *Leptospira interrogans*, *Leptospira borgpetersenii*, and *Leptospira kirschneri*. In dogs, leptospirosis is typically associated with infection by *L. interrogans* and *L. kirschneri*. More than 300 serovars, determined by the LPS structure, have been classified into 20 serogroups within these *Leptospira* species.^18,32^ Each serovar is adapted to a so-called maintenance or reservoir host, where the specific *Leptospira* spp. often causes mild to inapparent disease, induces a low antibody response, persists in the renal tubules, and is transmitted endemically within the hosts’ own species.^32,36,42^ In other nonadapted host species, incidental hosts, the serovar causes mild to severe clinical disease, induces high antibody titers, and the infectious organisms are eventually cleared.^12^ Worldwide, the serovar Canicola is maintained by dogs. In the USA, serovars Grippotyphosa and Pomona cause incidental clinical disease in dogs, whereas serovars Grippotyphosa and Bratislava are the most prevalent in Europe.^13^ Unique clinical signs, clinicopathologic abnormalities, and lesion pattern has been attributed to specific serovars.^15^

Vaccination is the method of choice to protect dogs from leptospirosis^12^ and the disease is transmitted by contact of injured skin or mucous membranes with water or soil contaminated by urine of the reservoir species, like rodents. The predominant clinical signs of acute leptospirosis are due to acute kidney injury, liver impairment, and hemorrhagic syndrome.^25,31,35^ Animals often die due to renal and hepatic failure.

The main histopathological findings in acute disease in the kidney are suppurative interstitial nephritis and tubular necrosis. In these lesions, leptospiral antigen is detectable. Glomerular changes are occasionally present, but tend to be discrete and nonspecific, such as mild membranoproliferative glomerulonephritis (GN). In chronic leptospirosis, interstitial nephritis with lymphocyte and plasma cell infiltration and fibrosis are seen.^1,4,9,12,19,33,36,38^

In Switzerland, however, over the past 20 years, a high number of suspect cases of canine leptospirosis presented with severe kidney disease with marked unusual glomerular changes, that is, exudative GN. In this retrospective study, the glomerular pathology is described and an association with leptospirosis is investigated. In addition, signalment, epidemiological data, clinical signs, and lesions in other organs are reported, which may assist in understanding the pathogenic mechanism, distribution, and associated factors of this condition.

## Material and Methods

### Case Selection

Included in this study were dogs that underwent postmortem examination in the Institute of Veterinary Pathology, Zurich and the Institute of Animal Pathology, Bern, Switzerland during a 20-year period between 1996 and 2015. The archives were searched for dogs that had a suspect or confirmed diagnosis of leptospirosis or that had a histopathological diagnosis of exudative GN. Exudative GN was defined as abundant extravasated erythrocytes and fewer neutrophils accompanied by marked fibrin exudation into the urinary space. Overall, 99 suspect canine leptospirosis cases were retrieved from the archives, 50 of which had a diagnosis of exudative GN. No exudative GN cases were found that were not leptospirosis suspects. The necropsy reports of the cases were reviewed, and the following data were evaluated where available: signalment, season, geographical location, clinical and laboratory data, and pathologic changes of all organs, in particular the lungs, liver, and kidney.

### Histology, Silver Stain

Formalin-fixed paraffin-embedded (FFPE) renal tissue blocks were retrieved from the archive and hematoxylin eosin stained slides were examined. Glomerular changes were described. Additional renal pathology, such as tubular necrosis and regeneration, were recorded as well as interstitial changes, including hemorrhage, edema, and suppurative or lymphoplasmacytic inflammation. Representative renal sections (*n* = 10) were stained with periodic acid-Schiff to highlight the glomerular basement membrane and phosphotungstic acid-hematoxylin for fibrin deposits.^6^

A silver stain (Warthin and Starry [W&S] ArtisanLink DAKO) was performed on the kidneys to demonstrate the presence of spirochetes. Where available from the necropsy reports, results of previous silver stains (W&S) on the liver were included.

### Immunohistochemistry

Immunohistochemistry (IHC) using rabbit anti-OMV2177 raised against LPS of *L. interrogans* serovar Copenhageni^35^ was performed in 30 cases on sections of liver and kidney and in 20 cases of kidney alone. The majority of renal samples that were negative or inconclusive by IHC using OMV2177 (*n* = 27) were tested with an antibody raised in rabbits against LipL32 (kindly provided by Dr Jarlath Nally, USDA Agriculture Research Service, Aimes, Iowa, USA), a major outer membrane protein present on all pathogenic Leptospira.

Two to three micrometer sections were cut from the paraffin blocks, mounted on positive charged glass slides, and dried over night at 37°C. The samples were then deparaffinized, rehydrated, and pretreated with acidic buffer (DakoCytomation Target Retrieval Solution Citrate pH 6 [10×], S2369) for 20 minutes at 98°C in a pressure cooker (Pascal DakoCytomation) for antigen retrieval. After every step, the slides were rinsed for 10 minutes with wash buffer (Dako Wash Buffer 10×, Dako S3006). The slides were then treated by a peroxidase block (Dako, S2023) for 10 minutes at room temperature (RT) and incubated for 30 minutes at RT with the primary antibody rabbit anti-OMV2177 at a dilution of 1:2000. As a secondary kit, the EnVision Rabbit was used (EnVision^+^Labeled Polymer System-HRP anti Rabbit, Dako K4003) for 30 minutes at RT. AEC (amino ethyl carbazol, Dako K3464) was used as chromogen for 10 minutes at RT, and slides were counterstained with hematoxylin. IHC for LipL32 was conducted on BOND-III Fully Automated stainer (Leica Microsystems Ltd, UK) using citrate buffer pH 6.0 (Bond TM Epitope Retrieval 1, Leica Biosystems AR9961) for 20 minutes at 100°C for antigen retrieval, 5-minute peroxidase block, and an antibody dilution of 1:1250 for 60 minutes at RT. Primary antibody labeling was detected with the Bond Polymer Refine Detection kit (Leica Biosystems DS9800), which includes polymer antirabbit poly-HRP-IgG, DAB (3, 3′diaminobenzidine) chromogen and hematoxylin counterstain. The kidney of a dog with known leptospirosis and moderate antigen content in the kidney and renal tissue of a rat experimentally infected with *L. interrogans* serovar Copenhageni were used as positive controls for both antibodies. Renal tissue from a dog with unrelated disease was used as negative control.

### Serology

The serological results (titer and serovar) were available from 25 cases. The test used was the microscopic agglutination test according to World Organization for Animal Health standards.^19,30^ Following serovars were tested: Autumnalis, Australis, Ballum, Bataviae, Bratislava, Canicola, Grippotyphosa, Hardjo, Icterohaemorrhagiae, Pomona, Tarassovi, and Sejroe.

### Polymerase Chain Reaction

As a different confirmatory test for pathogenic *Leptospira*, polymerase chain reaction (PCR) was conducted on 50 FFPE renal tissue blocks. For the PCR analysis, 40 μm sections were taken aseptically from each block and placed in a 1 ml PCR microcentrifuge tube in duplicate. DNA was extracted from the paraffin-embedded sections using a commercial kit (DNeasy Blood & Tissue Kit; Qiagen, Venlo, Netherlands). The pretreatment protocol for paraffin-embedded tissue as detailed in the instructions for use included washing steps using xylene and ethanol. Then, a PCR was performed using a Taqman probe with primers for LipL32 (Primer LipgrF2 [5′-CGCTGAAATGGGAGTTCGTATGATTTCC-3′]  and primer LipgrR2 (5′-GGCATTGATTTTTCTTCYGGGGTWGCC-3′)). An attempt was made to further identify the *Leptospira* species using a second PCR with a SYBR green probe by targeting *secY* as previously described.^2^ This PCR was conducted by A. Ahmed at the WHO/FAO/OIE and National Collaborating Center for Reference and Research on Leptospirosis, Amsterdam, Netherlands on FFPE renal tissues from 4 cases that had marked IHC reactions.

## Results

The presence of leptospiral antigen was confirmed in the kidneys of 37/50 dogs with exudative GN (Table 1). The ages of the affected dogs ranged from 2 months to 14 years (mean and standard deviation: 5.32 ± 4.07 years, median: 6 years), with 11 dogs younger than 6 months. The sex was recorded for 36 dogs with the majority being male (*n* = 21, 58%), of which 6 were castrated. Fifteen dogs were female (42%) with 6 being neutered.

**Table 1.** Results using W&S, IHC, and PCR for the detection of *Leptospira* spp. in renal tissue of 37 cases of exudative glomerulonephritis (GN).

Detection Method	Exudative GN (Total, *n* = 50)	
	Overall positive (*n* = 37)	Cases positive by more than one method
IHC	*n* = 32	
Ab OMV2177	*n* = 9	W&S, *n* = 9; PCR, *n* = 2
Ab LipL32	*n* = 23	W&S, *n* = 10; PCR, *n* = 2
	IHC negative (*n* = 18)	W&S, *n* = 2; PCR, *n* = 2
W&S	*n* = 21	IHC, *n* = 19; PCR, *n* = 2
PCR	*n* = 13	
	PCR alone (*n* = 5)	

From the 13 negative cases, 5 were only positive by W&S, implying leptospirosis.

Abbreviations: W&S, Warthin and Starry; IHC, immunohistochemistry; PCR, polymerase chain reaction.

Twenty-three breeds were represented, the most common being the Labrador retriever (*n* = 7) followed by mixed breed dogs (*n* = 6) and Golden retrievers (*n* = 2). Large breed dogs were most common (*n* = 16), followed by medium-sized dogs (*n* = 10) and small breed dogs (*n* = 4).

Most cases were from the canton of Zurich (*n* = 18) and the canton of Aargau (*n* = 7) followed by the canton of Bern (*n* = 4; Supplemental Fig. 1). The majority of cases occurred in late summer and early fall with 17 cases from July to August, 13 cases in September to October, and 3 cases in November. In 8 cases, no month was recorded.

The most common clinical signs were vomiting, which was occasionally bloody (*n* = 21) and icterus (*n* = 20). Diarrhea (*n*= 14), often associated with blood, anuria (*n* = 10), anorexia (*n* = 9), hematuria (*n* = 7), apathy (*n* = 7), and dyspnea (*n* = 5) were less frequent findings. Fever was only reported in 1 dog. When available (*n* = 12), hematology indicated anemia (*n* = 4) and/or thrombocytopenia (*n* = 9). Biochemistry results were available from 26 dogs. Renal azotemia was commonly reported (*n* = 19) and/or liver enzymes, including alanine aminotransferase, alkaline phosphatase, and total bilirubin were less often increased (*n* = 8).

### Pathological Changes, W&S, and IHC

Macroscopically, petechial to larger renal cortical hemorrhages were seen in 16 cases, 15 had no gross changes, and 6 had a swollen discolored kidney.

**Figure 1.**
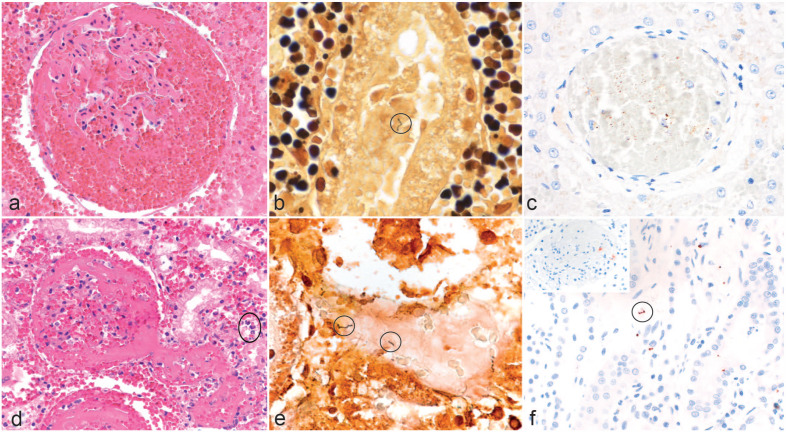
Histology, silver stain, and immunohistochemistry in canine leptospirosis cases with exudative glomerulonephritis. (a) Fibrin and abundant extravasated erythrocytes are visible in the glomerular tuft, mesangium, and the urinary space. Also, interstitial hemorrhage is present. Hematoxylin and eosin (HE). (b) A black leptospiral organism with the “Kleiderbügel” (cork-shrew) shape is present in a sloughed tubular epithelial cell and highlighted with an oval frame. Warthin and Starry (W&S). (c) Brown multifocal and pinpoint immunolabeling is present within a glomerulus obliterated by erythrocytes. Immunohistochemistry using anti-Leptospira LipL32. (d) Globally, the glomerulus is obscured by fibrin and abundant extravasated erythrocytes in the glomerular tuft, mesangium, and the urinary space. The supplying and draining vessels are occluded by fibrin thrombi. Periglomerular extravasated erythrocytes and acute tubular necrosis are visible with pyknotic nuclei and hypereosinophilic cytoplasm in the tubular cells admixed with few neutrophils (oval frame). HE. (e) Black argyrophilic leptospiral organisms are observed in the lumen of a necrotic tubule highlighted by oval frames. W&S. (f) Positive immunolabeling showing an intact spirochete in the tubular lumen (oval frame) and granular positivity of leptospires in the interstitium. Inset: A moderate number of red granules (leptospira antigen) can be seen in the glomerulus. Anti-leptospira immunohistochemistry using anti-OMV2177.

**Figure 2.**
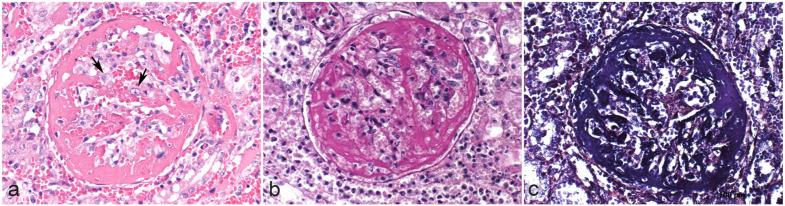
Histopathologic features of exudative glomerulonephritis in dogs with leptospirosis characterized by special stains. (a) Large amounts of fibrin admixed with fewer erythrocytes are obscuring the glomerulus and expanding the urinary space. The glomerular tuft is necrotic. Crescent formation (fibrocellular) is beginning and activated mesangial cells are present (arrows). The surrounding interstitium shows acute hemorrhage and fibrin exudation. Hematoxylin and eosin. The fibrinous character of the exudate can be confirmed by (b) *periodic acid-Schiff reaction and* (*c*) *phosphotungstic acid-hematoxylin stain*.

Microscopically, exudative GN affected more than 50% of the glomeruli (diffuse lesion) and most of the glomerular tuft was involved (global lesion) in all cases (*n* = 37). Exudative GN was characterized by large amounts of fibrin, abundant extravasated erythrocytes, and neutrophils in the glomerular tuft, mesangium, and mostly expanding the urinary spaces, compressing the glomerular tuft and ultimately obscuring the glomeruli. In some glomeruli, activated mesangial cells were visible. Some glomerular tufts were necrotic and early crescent formation (fibrocelullar) was evident in some glomeruli. The capillaries of the glomeruli were often dilated by erythrocytes. The afferent and efferent arterioles were often occluded by fibrin exudate. Periglomerular inflammation with extravasated erythrocytes and neutrophils was frequently visible (Figs. 1a, d, and 2a). Using a periodic acid-Schiff reaction, the fibrinous character of the exudate was confirmed and the basal membranes that could be assessed were not thickened (Fig. 2b). By means of phosphotungstic acid-hematoxylin staining the fibrinous character of the exudate was additionally confirmed (Fig. 2c). A summary of the additional histopathological findings in kidney, lung, and liver is shown in Table 2. Acute tubular necrosis with degeneration and/or necrosis of tubular epithelial cells, pyknotic nuclei, and hypereosinophilic cytoplasm, as well as sloughing of tubular epithelial cells into the lumen of tubules, was frequently seen. Tubular regeneration characterized by mitotic figures and/or double nucleated tubular epithelial cells was only occasionally observed. Acute multifocal to coalescing hemorrhages were seen in the interstitium in about two thirds of the cases. Interstitial nephritis was diagnosed in 29 cases. Infiltration of neutrophils (*n* = 19) was associated with varying amounts of lymphocytes and plasma cells in the interstitium in some cases (*n* = 6). Lymphoplasmacytic infiltrates alone were present in another 10 cases. Varying amounts of tubules contained bright eosinophilic homogeneous (protein) casts in 10 cases.

**Table 2.** Exudative glomerulonephritis in dogs seen in relation to other renal, lung, and liver histopathology in confirmed cases of leptospirosis.

Microscopic Changes	Exudative GN (*n* = 37)
Acute tubular necrosis	32 (86.5%)
Tubular regeneration	5 (13.5%)
Protein casts in tubules	10 (27%)
Suppurative interstitial nephritis	19 (51.4%)
Lymphoplasmacytic interstitial nephritis	16 (48.6%)
Interstitial hemorrhages	25 (67.6%)
Fibrinoid degeneration of small vessel walls	4 (11%)
Lung Lung hemorrhage	(*n* = 30) 27 (90%)
Liver Hepatocellular dissociation	(*n* = 33) 27 (81.8%)

Abbreviation: GN, glomerulonephritis.

Fibrinoid degeneration of the walls of smaller vessels in the interstitium was seen in 4 cases and thrombosis was present in another 2 cases. Lesions in the renal pelvis included mild, mostly lymphocytic, infiltrates (*n* = 6), or acute hemorrhage (*n* = 3).

Data on lung pathology were available for 30 cases. Multifocal variably extensive pulmonary hemorrhages, ranging from petechial to ecchymotic to lobar, were seen in 27 dogs (Table 2).

**Figure 3.**
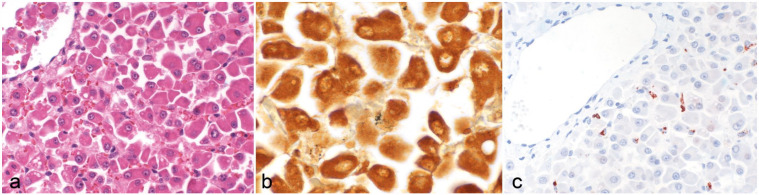
Histology, silver stain, and immunohistochemistry in the liver of canine leptospirosis cases. (a) Dissociation of hepatocytes is observed. Hematoxylin and eosin. (b) Black, argyrophilic fragments of leptospiral organisms are visible in the cytoplasm of few hepatocytes. Warthin and Starry. (c) Red-brown, granular leptospiral antigen labeling is seen in the cytoplasm of Kupffer cells. Immunohistochemistry using anti-OMV2177.

Livers were macroscopically unremarkable or slightly swollen; however, histologically, in 27 of 33 cases, diffuse hepatocellular dissociation with disruption of the cell cords and sinusoidal lining was seen. The hepatocytes were individualized with a rounded outline, and there were multifocal mitotic figures and occasional pyknotic nuclei (Fig. 3a). In 4 cases, there were multifocal random areas of hepatocellular necrosis and sinusoidal leukocytosis.

Overall, 21 cases had identifiable leptospira with W&S (57%). In 8 cases, the results remained questionable, with only small black fragments evident even after repeated staining, possibly due to degradation of the leptospiral organisms or of the marked inflammatory processes and hemorrhage obscuring the stain. In the kidney, the argyrophilic bacteria were mostly found in the proximal tubular epithelia. Most of them were degraded and only few had the typical cork-shrew morphology (Fig. 1b, e). Argyrophilic bacteria were found in the hepatocytes or sometimes sinusoids (most likely in Kupffer cells; Fig. 3b).

The presence of leptospira antigen was confirmed by IHC using the OMV2177 antibody in 9 cases (24%). Positive red-brown granular labeling was seen in the proximal tubular epithelial cells; cells in the glomeruli, most likely mesangial cells or admixed with the erythrocytes; cells located in the interstitium, for example, spindle-shaped cells interpreted as fibrocytes and macrophages; or macrophages in the vessels, mostly in the subcapsular veins. In general, only small amounts of labeling were associated with acute tubular necrosis. The labeling was mostly dot-like to granular and only visible in about 10% of the tubular structures. Minimal antigen was present in the cases with chronic interstitial nephritis, with seldom intact spirochetes being visible. In the glomeruli, a moderate amount of antigen was visible in some cases also as dot-like to granular labeling. The labeling was segmentally arranged in the glomeruli but only in approximately 20% of the glomeruli visible (Fig. 1f and inset). In the liver, labeling was observed mostly in Kupffer cells and sometimes in hepatocytes (Fig. 3c) as in contrast to the negative cases were no labeling was seen (Supplemental Fig. S2).

Twenty-three cases could be confirmed using the LipL32 antibody for IHC (62%). A similar labeling pattern was observed as described for OMV2177 (Fig. 1c). However, in addition, dense, granular labeling was found globally in up to 10% of glomeruli and in the apical borders of tubular epithelial cells in multifocal groups in about 10% of cortical tubules.

### Leptospirosis Serology

Data on the microscopic agglutination test were available in 25 cases and only 2 cases had a titer of 1:100 or more (12%). Serovars were only identified in one of these cases: *L. interrogans* serovar Icterohaemorrhagiae (1:800) and *L. interrogans* serovar Grippotyphosa (1:800).

### Polymerase Chain Reaction

A total of 13/37 cases (35%) had a positive PCR result. *L. interrogans* was identified using SybrGreen PCR in 1 of the 4 cases evaluated.

## Discussion

In our study, we describe exudative GN in dogs and demonstrate an association between the glomerular changes and an infection with *Leptospira* spp. The term exudative GN was mentioned by Bown (1977) in a clinical review of GN in dogs as one of the 3 GN classification types used by pathologists.^8^

Exudative GN was a frequent finding in dogs diagnosed with leptospirosis in Switzerland. Thirteen of the 50 cases of exudative GN could not be confirmed as leptospirosis by either IHC or PCR. However, the presence of intact W&S positive spirochetes in the renal tissue of 5 of these 13 negative cases, and the evidence of icterus, acute renal and hepatic failure, and hepatocyte dissociation in the majority of cases would support a similar etiology. Glomerular abnormalities ranging from glomerular hyperemia and mesangial matrix expansion to marked fibrinoid change, necrosis, or sclerosis have only been briefly mentioned before in a small number of dogs with *L. interrogans* Australis serogroup infection.^27^ Recognizing new diagnostic features of a zoonotic disease, which may present as a diagnostic challenge in its acute form, is of great importance to diagnostic pathologists.

Typically, published descriptions of acute leptospiral renal lesions in dogs and other species include acute tubular necrosis and/or interstitial or tubulointerstitial nephritis.^1,4,12,39^ These morphological changes were also observed in dogs with exudative glomerular lesions. In addition, typical hepatic involvement characterized by hepatocellular dissociation, single-cell necrosis, and mitotic figures^34^ was commonly seen in the dogs. These lesions are considered to be the result of leptospires infiltrating the space of Disse, then migrating between hepatocytes thereby detaching the intercellular tight junctions and disrupting the bile canaliculi.^29^ This results in the disruption of canalicular flow, causing intrahepatic cholestasis and jaundice.^29^ However, in a case series on acute fatal leptospirosis in young dogs, these hepatic changes were only accompanied by mild renal pathology.^33^ In addition, multifocal pulmonary hemorrhages, similar to the widespread hemorrhages of acute leptospirosis observed in dogs experimentally infected with serovar Pomona,^16^ was frequently observed in all study dogs. These, however, differed markedly in severity from the recently described leptospiral pulmonary hemorrhage syndrome in dogs^23^ and were therefore deemed unrelated to leptospiral pulmonary hemorrhage syndrome.

In the present cases of exudative GN, periodic acid-Schiff reactions revealed an intact and thin glomerular basement membrane, which may suggest that changes were caused by marked increase in vascular permeability within the glomerular capillaries rather than by circulating immune complexes. Still, circulating immune complexes cannot be ruled out without ancillary tests like immunofluorescence or electron microscopy. In addition, no intravascular thrombi, one of the key features of thrombotic microangiopathy, were observed in the vasculature of the main organs including the skin.^21^ Only single thrombi were present in medium-sized renal arteries in two cases with renal infarcts. Multifocal fibrinoid necrosis of small renal arterial vessels in the interstitium and pelvic region was seen in 4 cases, which has been reported in dogs with acute fatal leptospirosis.^33^ The absence of extensive thrombotic vascular changes in the study dogs would therefore rule out the main differential diagnosis of hemolytic-uremic syndrome^20^ and idiopathic cutaneous and renal glomerular vasculopathy.^21^ The marked increase in vascular permeability seen in exudative GN could be linked to 2 of the main hypothesized mechanisms in the pathogenesis of leptospirosis, which are (1) the disruption of endothelial cell adherens junctions, which are mediated by VE-cadherin, an important receptor for pathogenic leptospires and (2) the alteration of the cell membrane, leading to altered permeability.^10,27^ Unfortunately, attempts to further characterize exudative GN by electron microscopy failed due to poor quality of the archived FFPE tissue blocks. The only other crescentic proliferative GN in animals has been described in pigs with porcine dermatitis nephropathy syndrome, which has been postulated to be an immune-complex disease characterized by fibrino-necrotizing GN and systemic vasculitis linked to porcine circovirus-2.^3,9,37^

As described in the literature, in the present study, there was a higher proportion of male dogs compared with female dogs. Increased outdoor activity and canine male specific behavior, including sniffing and licking of urine, potentially favoring dog-to-dog transmission, have been suggested to explain the male overrepresentation.^25^ Also, most cases occurred between April and November and peaked in late summer/early autumn associated with higher temperatures and higher rainfall. A large variety of breeds were affected, as previously reported.^5,11,25,32,33^ In the present study, puppies were overrepresented, as described before.^25^ Overall, dogs with exudative GN showed similar sex, age, and seasonal predispositions when compared with the suspected leptospirosis cases without exudative GN mentioned in the case selection and to a report on incidence of canine leptospirosis in Switzerland during the same study period.^25^

In the present study, only FFPE tissues were available for confirmatory testing; therefore, IHC and PCR were used for the diagnosis. The W&S silver stain is not specific for *Leptospira*, but it is useful to identify spirochetes in tissue especially in chronically infected carrier animals that have large amounts of intact leptospires in the tubular lumen.^41^

The use of 2 antibodies raised against different Leptospira antigens markedly increased the detection rate when using IHC in the present study. Here, the OMV2177 antibody raised against LPS of *L. interrogans* serovar Copenhageni was used with an expected strong reactivity to other *L. interrogans* serovars, but with likely limited reactivity when used on serovars of other serogroups. Varying immunoreactivity to the same antiserum likely reflects the relative expression of the leptospiral antigens against which the antiserum is prepared.^7,41^ Therefore, the low detection rate of only 24% using OMV2177 antibody suggests that other *Leptospira* spp. are involved in the etiology of this glomerular pathology. In contrast, the LipL32 antibody targets a single protein on the outer membrane expressed by all pathogenic leptospires. While this antibody can detect all serovars and greatly enhanced the sensitivity of the IHC in this study, it is much more dependent on the expression of a single protein and therefore the number of bacteria present in the sample. It can identify intact leptospires colonizing the renal tubules, as seen in our cases, but may be limited in the detection of fragments of leptospires phagocytosed by macrophages, as seen in the liver. The IHC positivity in Kupffer cells confirmed the presence of leptospiral antigen suspected by W&S and has been reported in humans with fatal leptospirosis using an antibody targeting the same epitope LipL32.^22^

Limited antigen recognition could have been due to the large amounts of extravasated erythrocytes, neutrophils and fibrin that could have degraded and obscured the antigen. In addition, the best reactivity in IHC is with an antibody that is raised against the LPS of the serovar with which the animal is infected. Furthermore, the detection of *Leptospira* postmortem can be optimized by testing multiple organs (lungs, liver, and kidney).^32^ This, however, was not the case in the present study where examining the liver by IHC and W&S gave more questionable results than the kidney and did not provide additional information.

The PCR for *Leptospira* spp. was only positive in 35% (13/37) of the cases. As the PCR was conducted on FFPE tissues, this poor result was likely due to the limited amount of nucleic acid present, or the age and fixation of samples. Retrospective differentiation of *Leptospira* spp. using PCR^2,14^ on FFPE tissues was attempted. One of 4 cases of exudative GN tested was identified as *L. interrogans* using the SybrGreen PCR. Poor DNA yield retrieved from FFPE tissues hampered further analyses.

In summary, the decreased reactivity with OMV2177 antibody raised against LPS of *L. interrogans* in IHC and the low proportion of samples positive for *L. interrogans* by *secY* PCR in cases of exudative GN compared with classical leptospirosis may suggest the involvement of a species different from *L. interrogans*.

Dogs included in this study died or were euthanized after a short severe disease progression (up to 10 days), often associated with typical clinical signs.^24,36^ Antemortem diagnosis of leptospirosis generally relies on the detection of high serum antibody titers using the microscopic agglutination test, which detects serogroups and serovars.^36^ In the present study, antibodies were only detected in 1 of 25 cases, which is likely due to the acute short course of disease where antibodies had not been formed.^36^ Here, two serovars, *L. interrogans* serovar Icterohaemorrhagiae and Grippotyphosa, could be identified in one case, which makes it difficult to predict the infecting serovar.^28^

Common serovars associated with canine leptospirosis in Europe are Grippotyphosa, and Bratislava.^12^ In Switzerland, serologic evidence points to infections with the serovars Australis and Bratislava followed by Copenhageni, Canicola, Grippotyphosa, Pomona, Autumnalis, and Icterohaemorrhagiae.^11^

Circumstantial evidence supports the involvement of a specific serovar in the pathogenesis of exudative GN. The species *L. kirschneri serovar* Grippotyphosa, which is included in the new vaccine, for example, is known to cause renal lesions like interstitial nephritis and renal tubular degeneration.^16^ Since the introduction of the new vaccine in Switzerland, cases of leptospirosis have been markedly reduced.^11,12^ However, for *Leptospira* species, identification culture of urine or renal tissues of affected dogs would be necessary.

## Conclusion

In this study, exudative GN associated with acute leptospirosis in dogs was described in detail and determined as a frequent characteristic finding of the disease. Acute fatal canine leptospirosis may present a diagnostic challenge to the pathologist and this new feature will help to reach a diagnosis. The underlying pathogenesis remains unclear but the geographic location and a *Leptospira* species other than *L. interrogans* may be linked to the disease. Age, breed, sex, and season were not identified as risk factor for exudative GN. Clinical signs as well as pathology in tissues other than glomeruli did not differ between dogs with exudative GN and what has been reported in the literature in cases of leptospirosis without GN.

## Supplemental Material

sj-pdf-1-vet-10.1177_03009858231207020 – Supplemental material for Exudative glomerulonephritis associated with acute leptospirosis in dogs Supplemental material, sj-pdf-1-vet-10.1177_03009858231207020 for Exudative glomerulonephritis associated with acute leptospirosis in dogs by Monika Hilbe, Horst Posthaus, Giulia Paternoster, Simone Schuller, Michelle Imlau and Hanne Jahns in Veterinary Pathology
