# Supplementary material for: Exudative glomerulonephritis associated with acute leptospirosis in dogs
Source: Vet Pathol. 2023 Oct 29;61(3):453–61. doi: 10.1177/03009858231207020 (PMC11067394; doi:10.1177/03009858231207020)

Supplemental Materials

Exudative glomerulonephritis associated with acute leptospirosis in dogs

Monika Hilbe, Horst Posthaus, Giulia Paternoster, Simone Schuller, Michelle Imlau, Hanne Jahns

Supplemental Figure S1. Geographic distribution from all retrieved (n=99) Swiss leptospira cases.

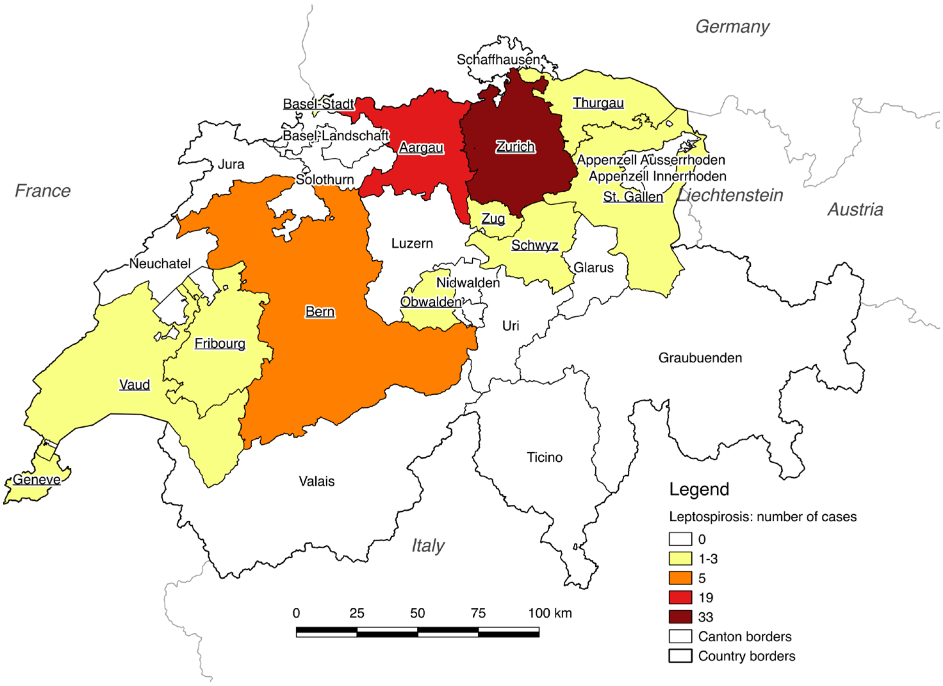

Supplemental Figure S2. Histology, silver stain, and immunohistochemistry in the negative liver of canine leptospirosis cases. **a)** Dissociation of hepatocytes is observed. Hematoxylin and eosin. **b)** No black, argyrophilic fragments of leptospiral organisms are visible in the cytoplasm of hepatocytes or Kupffer cells. Warthin and Starry. **c)** The immunohistochemistry is negative and shows no labeling in the cytoplasm of Kupffer cells. Immunohistochemistry using anti-OMV2177.

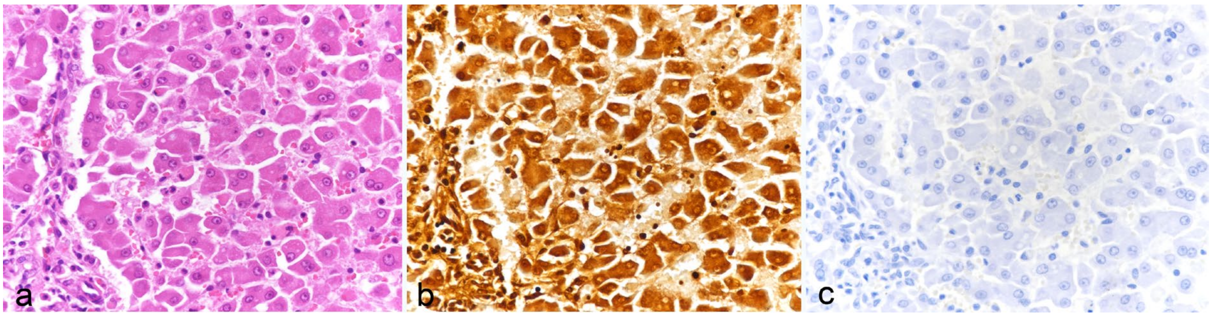

Supplement: sj-pdf-1-vet-10.1177_03009858231207020 – Supplemental material for Exudative glomerulonephritis associated with acute leptospirosis in dogs [file sj-pdf-1-vet-10.1177_03009858231207020.pdf]
